# Citizen crowds and experts: observer variability in image-based plant phenotyping

**DOI:** 10.1186/s13007-018-0278-7

**Published:** 2018-02-09

**Authors:** M. Valerio Giuffrida, Feng Chen, Hanno Scharr, Sotirios A. Tsaftaris

**Affiliations:** 1 School of Engineering, Institute of Digital Communications, The University of Edinburgh, Edinburgh, EH9 3FB UK; 2 IMT School For Advanced Studies Lucca, Piazza San Francesco, 19, 55100 Lucca, Italy; 3 Institute of Bio- and Geosciences (IBG), IBG-2: Plant Sciences, Forschungszentrum Jülich GmbH, Wilhelm-Johnen-Straße, 52425 Jülich, Germany

**Keywords:** Phenotyping, Image-based, Observer, Agreement, Variability, Crowdsourcing, Citizen-science

## Abstract

**Background:**

Image-based plant phenotyping has become a powerful tool in unravelling genotype–environment interactions. The utilization of image analysis and machine learning have become paramount in extracting data stemming from phenotyping experiments. Yet we rely on observer (a human expert) input to perform the phenotyping process. We assume such input to be a ‘gold-standard’ and use it to evaluate software and algorithms and to train learning-based algorithms. However, we should consider whether any variability among experienced and non-experienced (including plain citizens) observers exists. Here we design a study that measures such variability in an annotation task of an integer-quantifiable phenotype: the leaf count.

**Results:**

We compare several experienced and non-experienced observers in annotating leaf counts in images of *Arabidopsis Thaliana* to measure intra- and inter-observer variability in a controlled study using specially designed annotation tools but also citizens using a distributed citizen-powered web-based platform. In the controlled study observers counted leaves by looking at top-view images, which were taken with low and high resolution optics. We assessed whether the utilization of tools specifically designed for this task can help to reduce such variability. We found that the presence of tools helps to reduce intra-observer variability, and that although intra- and inter-observer variability is present it does not have any effect on longitudinal leaf count trend statistical assessments. We compared the variability of citizen provided annotations (from the web-based platform) and found that plain citizens can provide statistically accurate leaf counts. We also compared a recent machine-learning based leaf counting algorithm and found that while close in performance it is still not within inter-observer variability.

**Conclusions:**

While expertise of the observer plays a role, if sufficient statistical power is present, a collection of non-experienced users and even citizens can be included in image-based phenotyping annotation tasks as long they are suitably designed. We hope with these findings that we can re-evaluate the expectations that we have from automated algorithms: as long as they perform within observer variability they can be considered a suitable alternative. In addition, we hope to invigorate an interest in introducing suitably designed tasks on citizen powered platforms not only to obtain useful information (for research) but to help engage the public in this societal important problem.

## Background

This community is well aware of the importance of measuring a plant’s phenotype and its modulation due to environmental and genotypic variations. Scientists have been observing plants directly, measuring phenotyping traits manually for years. Whilst this method is labour-intensive and time consuming, it is also prone to errors [1, 2]. Recently, image-based phenotyping by coupling imaging and automation has created a revolution on how we observe (and can potentially quantify) such phenotypic variation, in the hope of reducing the phenotyping bottleneck [3–5]. Without a doubt this potential has spurred a great interest in the imaging of plants at various levels of scale, above or below ground level, in the optical or hyper-spectral spectrum in 2D or 3D [6, 7].

However, the ability to extract actionable information from image data, that will lead to the full realization of this revolution, is still considered a hard task [8]. It is the complexity of some of the tasks involved that have now created a new bottleneck: lack of appropriate software solutions able to effectively analyze such data [9]. The community has reacted swiftly by placing significant emphasis in the design of new algorithms and the release of software (for example see the collection of http://www.plant-image-analysis.org and [10]). More recently, open datasets [11–13] have allowed not only the ability of experts within the community to evaluate algorithmic performance on key phenotyping tasks, such as leaf segmentation and counting, but also enabled image computing experts new to plant phenotyping to enter this exciting field [14–18]. Unsurprisingly, many of the new methods rely on machine learning, a technology that has the potential to transform how phenotyping discovery from images can occur in the future [19, 20], as also recently demonstrated [15, 16, 21]. Even though its potential is well-known, machine learning algorithms do require data to learn from, which typically need to be annotated by expert observers when domain-specificity is required. The performance of algorithms is bounded to the precision of observers. Naturally this raises the question *how precise are the experts on a given task?*

In the medical community, variability among observers is known to exist and has been accepted [22]. Also experts in plant breeding, diseases, and taxonomy agree that variability exists [23–25]. For example, several studies [26–28] have been used as de-facto references for discussing rater disagreement when visually scoring leaf diseases on the basis of scales. At the same time they have become motivating references advocating that image analysis systems can help reduce (rater) variation [29]. They have been also perused in advocating for the use of digital imaging itself as opposed to on site surveys with rating scales [30]. Even the image-based phenotyping literature has been perusing these works [30, 31]. However, an extensive literature review has not found a comparison of raters on visually *quantifiable* traits or phenotypes.

One such integer-quantifiable phenotype is counting the number of leaves (or fruits, flowers). Leaf count can be used to describe the growth status of a plant [32], and is obviously closely related to plastochron or phyllochron [33–35] and can be used to assess plants’ reactions to stress [34, 36]. Herewith lies a key difference: the count as a phenotype has a physical ‘ground truth’ which visual scales are not capturing and are not suited for. To this day, no such direct evaluation of observer agreement in leaf counting exists and to the best of our knowledge in the broader sense of image-based phenotyping of quantifiable phenotypes.

**Fig. 1:**
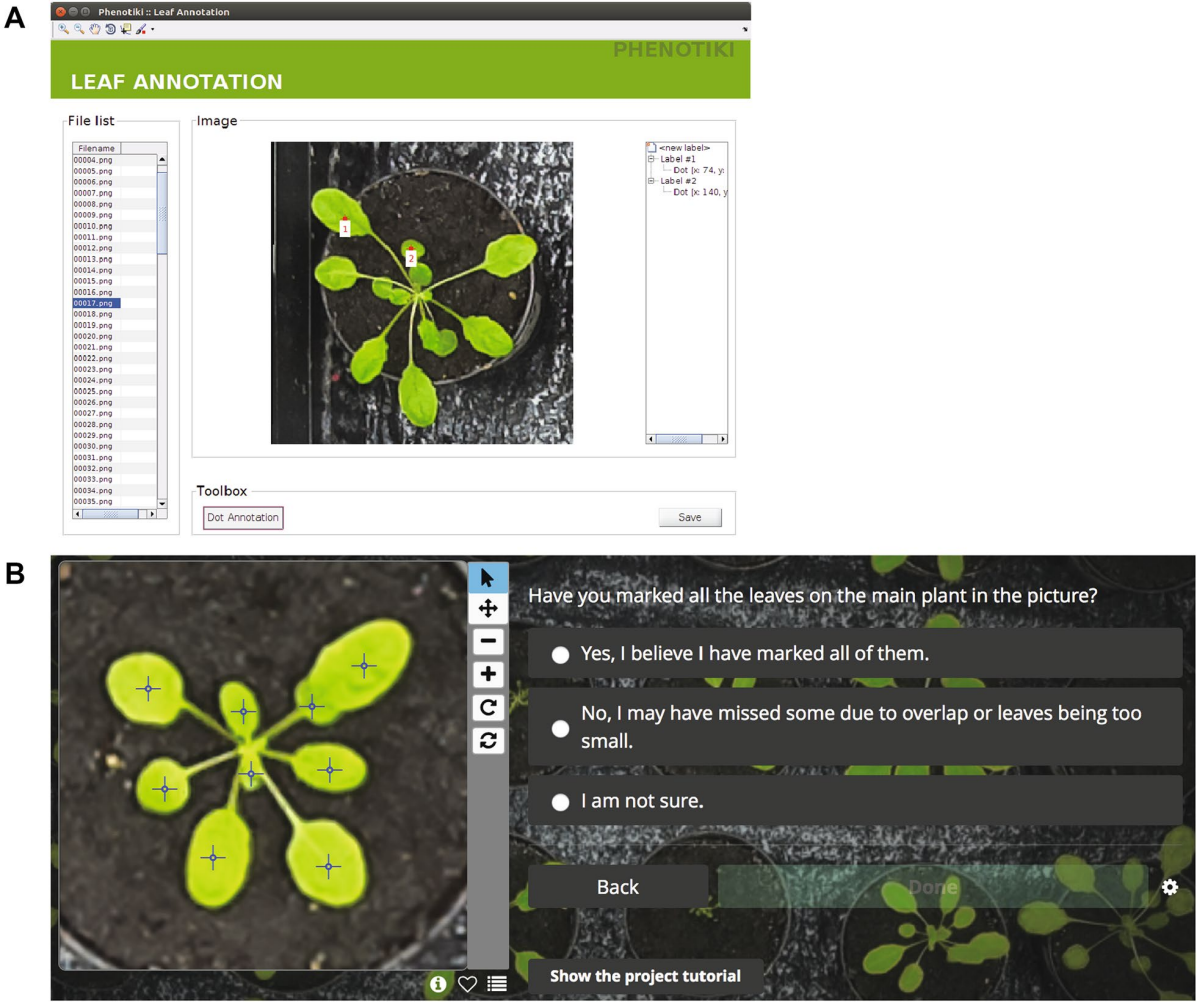
Annotation tool. Screenshots of the annotation tool and the web-page seen by users. **A** Screenshot of the customized, yet simplified, version of the leaf annotation tool in [21]. **B** An excerpt of the Zooniverse site used here showing annotations and the (single-choice) confidence question

**Fig. 2:**
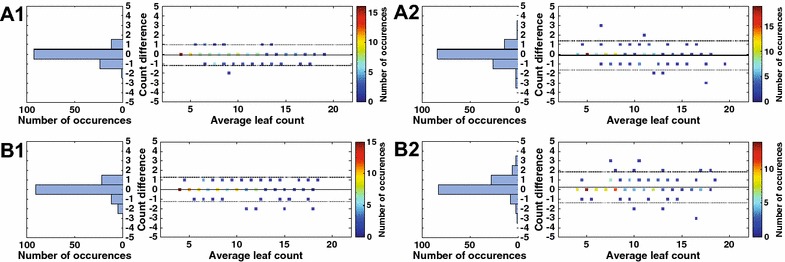
Intra-observer variability. **A** Intra-observer variability of experienced (left: **A1**) or non-experienced (right: **A2**) observers in RPi. **B** Influence of the tool in intra-observer measurements in experienced (left: **B1**) or non-experienced (right: **B2**) observers in RPi

Clearly, counting objects, here leaves, is a task generally doable even by non-experts without detailed explanations. This may not be true for other, maybe visually harder, phenotyping tasks. However, even though counting plant organs might seem an elementary task, many factors may result in different values among observers, such as severe occlusions, small objects in the scene, low camera resolution, as well as mental fatigue of the annotators.

Estimating observer variability is crucial because it primarily allows us to put bounds on effect sizes and devise annotation strategies that minimize annotation effort (e.g. by splitting annotation effort among many observers). At the same time, by evaluating agreement comparing experienced (expert) and non-experienced (non-expert) observers we can evaluate the potential of using non-experts for simple well-defined annotation tasks. In addition, it allows us to put the performance of algorithms in comparison to intra- or inter-observer variation and assess how close we are to achieve human performance. It may even permit us to devise different algorithmic approaches that learn despite the presence of disagreement [37, 38].

Equally exciting is the potential to explore how the use of common citizens can be used to not only annotate data for machine learning but as being part of a phenotyping experimental pipeline. The introduction of Amazon Mechanical Turk (AMT, https://www.mturk.com/) that permits the use of humans (via fee) in solving computer based microtasks in combination with annotation frameworks (e.g. LabelMe [39]) has led to an explosion of the potential use of crowdsourcing—a term was coined by Jeff Howe in 2006 [40]. It has been used for a variety of tasks already even for plant research e.g. http://photonynq.org. However, there have been ongoing debates as to how one can control the quality of outcomes because in principle, crowdsourcing allows ‘anyone’ to contribute. More recently, citizen-powered platforms, where volunteers participate to help with a task, as opposed to receiving a reward (a payment in real [AMT] or virtual money [Gamification]), have received particular attention by many researchers. One such popular platform, *Zooniverse* (http://www.zooniverse.org), allows researchers to build projects to collect data from thousands of people around the world, in order to support corresponding research. Several exciting projects have used the platform already: for example, Arteta et al. [41] used the data from a penguin watch project to automatically count penguins in the wild.

**Fig. 3:**
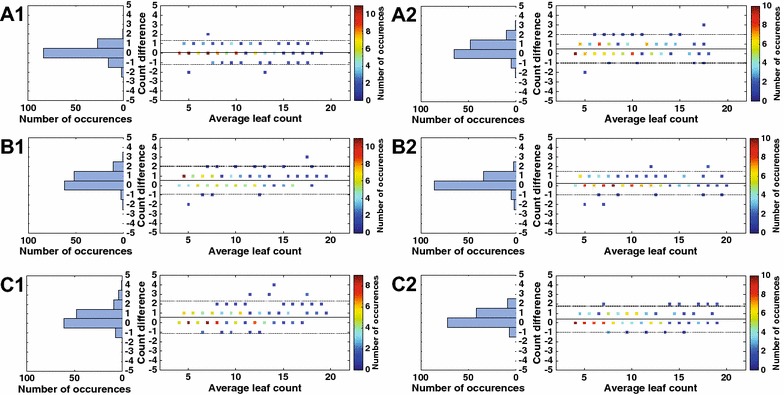
Inter-observer and influence of resolution. **A** Inter-observer variability among experienced (left: **A1**) or non-experienced (right: **A2**) observers in RPI; **B** same as in **A** but in Canon data; **C** Variability of experienced (left: **C1**) or non-experienced (right: **C2**) observers when comparing counts of the same observer in RPi and Canon data

**Fig. 4:**
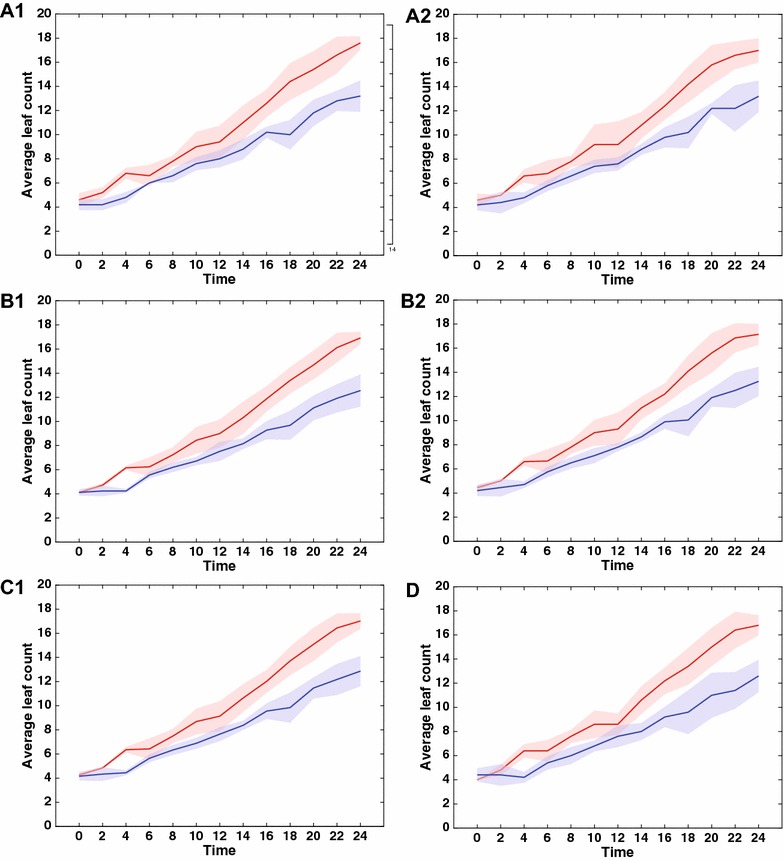
Average longitudinal counts. Average longitudinal count curves (solid) of the two cultivars [red: *col-0*; blue: *pgm*] and 1 standard deviation (shaded area), shown in **A** relying on a single experienced (left: **A1**) or non-experienced observer (right: **B1**); **B** relying on all experienced (left: **B1**) or non-experienced (right: **B2**) observers; **C** relying on all together; and in **D** relying on the consensus citizen

In this paper we aim to estimate observer agreement with a simple, yet expertly designed, image-based observational study. We select images of *Arabidopsis Thaliana* (taken from a dataset in the public domain [11]) and ask several observers to count leaves using a variety of setups in a controlled fashion. At the same time, we included the same images within a larger citizen-powered research project that runs on Zooniverse. Specifically, we aim to assess whether:

1. variations exist between the same observer (intra-observer);
2. computer-aided counting, using a specifically designed annotation tool, helps to reduce variability compared to straight-forward visual observation;
3. observers differ from each other (inter-observer);
4. higher resolution reduced observer variability;
5. observer variability has any statistical influence in separating a cultivar of known different leaf growth w.r.t. wild-type;
6. time needed for annotations depends on expertise;
7. we can simulate the effects of randomly sampling from an observer population on statistical inference;
8. counts from a citizen-powered study can be used for phenotyping; and
9. a recent ML algorithm that predicts leaf count from plant images performs within the variation of observers.

We address these points one by one in this order in the “Results” section.

**Fig. 5:**
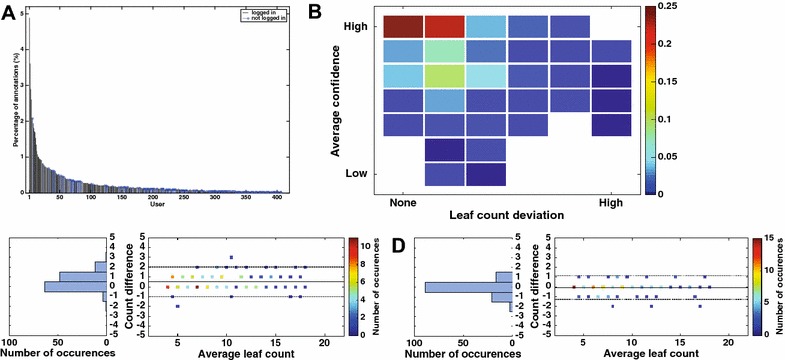
Citizen distribution and variability. **A** Number of images annotated per user (citizen); **B** Relationship between leaf count variation and average user confidence per plant; **C** Variability between the consensus citizen and the reference observer; **D** Variability between the consensus citizen and a random selection of counts (from the 3 available per-plant)

## Methods

We recruited 10 annotators: 5 who have experience with image-based plant phenotyping (shorthanded below as ExP) and 5 who do not have experience with phenotyping but yet have experience with images (shorthanded hereafter as NExP) to annotate a subset of the *Arabidopsis* dataset in [11]. Specifically, each annotator had a set of different tasks to accomplish using visual tools or simple observation designed to assess the influence of the factors considered in this study (see background above). Details of the approach taken are provided below.

### Employed image data

The data used in this study have been collected using an affordable imaging setup that used a Raspberry Pi camera, but also an optical zoom camera that offered a higher effective resolution [21]. Images of two cultivars were selected (the wild-type *col-0* and *pgm*), 5 replicates each every other day at 8am (i.e. every 48 h). *pgm* is known not to be able to accumulate transitory starch due to a mutation in the plastidic isoform of the phosphoglucomutase, which is required for starch synthesis and overall is known to be smaller than the wild-type [42]. Furthermore, *pgm* was recently shown to produce new leaves at a pace lower than wild-type [21]. Thus, we knew a priori that these cultivars should show differences in a longitudinal assessment of leaf count. The sampling frequency chosen (every 48 h) results in 13 time points per each plant, providing 130 images overall for annotation. This sampling frequency was chosen after statistical power analysis on the sample size of an ANOVA experiment [43] drawing effect sizes reported in [21].

**Table 1:** Measurement of agreement between experienced and non-experienced observers

	DiC ↓	|DiC| ↓	MSE ↓	*R*^2^↑	Alpha ↑
*Intra-observer (RPi) tool*					
Experienced [The reference observer]^a^	0.10 (0.54)	0.29 (0.47)	0.307	0.980	0.987
Non-experienced	0.13 (0.77)	0.42 (0.65)	0.600	0.960	0.981
*Tool versus visual (RPi)*					
Experienced	0.00 (0.64)	0.33 (0.55)	0.415	0.970	0.986
Non-experienced	0.23 (0.82)	0.46 (0.71)	0.730	0.950	0.977
*Inter-observer (RPi) tool*					
Experienced	0.07 (0.65)	0.37 (0.53)	0.423	0.974	0.980
Non-experienced	0.49 (0.76)	0.60 (0.67)	0.815	0.962	0.962
*Inter-observer (Canon) tool*					
Experienced	0.55 (0.74)	0.63 (0.68)	0.861	0.969	0.959
Non-experienced	0.23 (0.63)	0.37 (0.56)	0.450	0.977	0.976
*Intra-observer across resolution (RPi and Canon) tool*					
Experienced	0.57 (0.87)	0.68 (0.79)	1.100	0.950	0.965
Non-experienced	0.40 (0.70)	0.51 (0.62)	0.650	0.973	0.977
*Citizens inter-observer (RPi) zooniverse*					
Experienced versus consensus (average)	0.53 (0.77)	0.62 (0.69)	0.869	0.962	0.960
Experienced versus consensus (max)	0.08 (0.82)	0.45 (0.69)	0.684	0.957	0.971
Consensus (average) versus sing. random	0.00 (0.78)	0.42 (0.65)	0.607	0.960	0.970

For shorthand definitions see text. For DiC and |DiC| average and standard deviation are reported. Note that these correspond also to bias and limits of agreement (when standard deviation is multiplied by 1.96) of the Bland–Altman plots reported. $\downarrow$ means lower is better, whereas $\uparrow$ the opposite

^a^This experienced observer is noted and used as the reference observer for the remaining analysis throughout the paper

Images were cropped such that a plant appears centered in the field of view. Plant images from the Raspberry Pi camera had an effective resolution of 300 × 300 pixels (hereafter shorthanded as *RPi*), whereas the ones from the camera with movable optics had 470 × 470 pixels (shorthanded as *Canon*). In addition, to properly test intra-observer variability eliminating as much as possible effects of visual memory, a copy of all images was created, where images were artificially transformed by random 90°, 180°, 270° rotation or horizontal/vertical flip. These transformed datasets are shorthanded as *RPi’* and *Canon’*. Data within each set were randomized to break temporal consistency and within genotype associations and to satisfy an identically independently distributed (IID) data source design.^1^ Dataset names were obscured as A (RPi), B (Canon), C (RPi’), and D (Canon’) such that observers were blinded to what the sets meant and reduce possible bias in ratings.

### Study design

A customized graphical user interface, based on the annotation tool in Phenotiki,^2^ was specifically designed for this study [21, 44]. The tool prompted the user to select a dataset for annotation (from A, B, C, D) and the selected list of images was automatically loaded. For each image, the observer could place dot annotations marking every leaf they could identify. Critically dots remained visible throughout a plant annotation helping the annotator keep track of visited leaves. When the observer was done, they could proceed to the next plant. Zoom and pan functionality were available to help observers visualize scenarios such as small emerging leaves and occlusions. Annotation timing was recorded but observers were not aware of this fact. Annotation timing (per plant) was calculated as the time elapsed from the first and last leaf annotation for a given plant. An example of the interface seen by users is shown in Fig. 1A.

Experienced (with image-based plant phenotyping) and non-experienced observers were recruited to participate in this observational study. They were provided with a description of the purpose of the study, and were asked to consent to participate in the study. They were shown a guide and an introduction to the annotation tool to ensure a common baseline. Specifically, we showed them examples of good plant annotations, where they were asked to mark leaves at the center of the leaf blade (or the most visible area in case of severe overlap). Each observer was assigned two or more of the datasets to rate and count leaves. The order of the datasets shown was randomized and never of the same orientation (e.g. if one was shown A the next dataset would be C or D) to minimize effects of memory. To further reduce memory effects a 10 min break was enforced between annotation tasks.

**Table 2:** F and *p* values for the ANOVA tests corresponding to the plots in Fig. 4

	Sum sq.	F	*p* value
A single ExP	47.816	43.775	0.000167
A single NExP	47.170	30.017	0.000588
All ExP	56.264	34.661	0.000367
All NExP	49.533	29.116	0.000649
All observers	53.219	32.280	0.000464
Consensus citizen (average)	66.923	19.044	0.0024
Consensus citizen (max)	76.855	23.713	0.0012

Only time*cultivar interaction is shown corresponding to the factor of interest (longitudinal trend). Results with ‘All’ and consensus citizen average (or max) across per-plant observations

Some observers were asked to rate the images also without the use of the tool but recorded leaf counts in a spreadsheet after shown an image.

Time to complete each set was recorded in addition to the times recorded by the tool itself (see annotation timing above).

### Citizen-powered study

The A data (RPi) were included as part of a larger citizen-powered study (“Leaf Targeting”, available at https://www.zooniverse.org/projects/venchen/leaf-targeting) built on Zooniverse (https://www.zooniverse.org/). Using the Zooniverse application programming interface (API), an annotation work-flow was designed that showed an image to a user via a web browser. The users (random visitors) were asked to view a tutorial on how to annotate leaves. The task essentially involved placing a dot annotation on each leaf, thus retaining the characteristics of the interface used in the fully controlled study described previously. Users could as well zoom in and out and delete dot annotations. Users were also asked to answer a question after each plant was annotated as to their confidence in having annotated all leaves (encoded as Yes: 3, Not sure: 2, Missed leaves: 1). An example of an annotated image along with the interface and questions seen by the users are shown in Fig. 1B. We note that the users have the option to log in to the platform and also to comment about images where they can discuss issues related to the image or the task in general. We set the work-flow to repeat the same image 8 times after at least all images have been annotated 3 times; images for annotation are shown at random and thus annotations can be treated as IID and the same image is not rated by the same user. The system exports complete information for each annotated image such as image ID, user name (or unique IP), time, the locations and number of dots, and the response to the confidence question.

**Table 3:** A simulated citizen-powered experiment. *p* values corresponding to an ANOVA test randomizing the number of observations available per each plant at a specific time point

	*K*	Min	Max	Mean	Std	Kurtosis
Any	1	0.00003	0.00819	0.00124	0.00113	10.34
Any	2	0.00002	0.00729	0.00120	0.00112	8.98
Any	3	0.00010	0.00235	0.00061	0.00032	6.49
ExP only	1	0.00000	0.00726	0.00102	0.00103	9.58
ExP only	2	0.00004	0.00306	0.00057	0.00040	9.29
ExP only	3	0.00008	0.00150	0.00047	0.00021	5.35
NExP only	1	0.00008	0.00378	0.00100	0.00065	5.71
NExP only	2	0.00023	0.00174	0.00078	0.00028	3.49
NExP only	3	0.00033	0.00124	0.00069	0.00015	3.19

Process is repeated sampling from any of the observers (i.e. the sampling may contain a mix of experienced and non-experienced observers) or only from experienced (ExP) or non-experienced (i.e. NExP) ones

### Statistics and evaluation metrics

A variety of descriptive and summary statistics as well as several statistical methods were used to evaluate agreement in the controlled experiment. We note that in the case of discrete counts and heavily zero inflated differences (when comparing counts between observers) many of the common statistics and visualization methods can lead to misinterpretations. Thus, between a reference observer $(X_R)$ and one of the other observers $(X_o)$, we adopted:

- *Difference in count (DiC)* mean and standard deviation of difference between $X_R$ and $X_o$. [Zero is best.]
- *Absolute difference in count (|DiC|)* mean and standard deviation of absolute difference between $X_R$ and $X_o$. [Zero is best.]
- *Mean squared error (MSE)* squared difference between $X_R$ and $X_o$. [Zero is best.]
- *Coefficient of determination *(*R*^2^) the proportion of the variance in $X_R$ that is predictable from $X_o$. [One is best.]
- *Krippendorff’s alpha (alpha)* a chance-adjusted index of inter-observer agreement [45]. We used the mALPHAK implementation in Matlab [46] treating counts as a ratio scale variable comparing $X_R$ and $X_o$. [One is best.]

The first four metrics were adopted since they have been used to compare counting algorithms on the basis of challenge data [14].

To visualize agreement between pairs of observers we used a modified version of the Bland–Altman (BA) plot [47] in conjunction with the histogram of count differences. For the BA plot, we plot color labelled squares with square color varying according to how many points agree on the same coordinates. This is necessary since we observed that in scatter plots of discrete quantities, points will overlap misrepresenting the true distribution of the data.

**Table 4:** Algorithmic leaf counting results obtained using the method in [15]

	Algorithm versus annotator	Algorithm versus annotator	Annotator versus reference
	Training error	Testing error	Inter-observer error
DiC ↓	0.00 (1.07)	− 0.04 (1.31)	0.21 (0.75)
|DiC| ↓	0.61 (0.88)	0.88 (0.96)	0.46 (0.62)
MSE ↓	1.163	1.700	0.600
R^2^↑	0.933	0.895	0.964

Four metrics are reported. We first compare between the algorithm and the 728 images in the training set (ie. how well the algorithm learns). Then we compare how well the algorithm predicts counts on a testing set of 130 images (also used in this study) comparing the algorithm with the counts of the annotator (that also was involved in deriving annotations for the training set). Lastly we compare the annotator (the data of which we used to train the algorithm and was not involved in this study) with the reference observer used throughout in this study

Finally, while evaluating agreement is interesting on its own, we also considered an application-driven measure of agreement by estimating a mixed effect repeated measure two way ANOVA on count data as employed in [21] for the two cultivars. By this, essentially we test whether any observable differences exist in between cultivar longitudinal trends obtaining average counts using a different set of observers. We treated subject ID (i.e. the replicate) as a random effect whilst all other as fixed effects. To not over-inflate degrees of freedom we treated time as a continuous predictor. Of particular interest is the interaction term between time and cultivar (cultivar*time hereafter), since this is the term that tests longitudinal differences between the cultivars.

## Results

### Intra-observer variability

We assessed this via a second reading from the same observer using the tool. In Fig. 2A we plot histograms and Bland–Altman (BA) plots for two observers on the datasets A, C (ie. same as A but with geometric changes). Considering also the corresponding rows in Table 1, we can see that intra-observer agreement overall is excellent, with the NExP observer showing slightly higher variation (higher standard deviation) and decreased agreement (alpha) compared to ExP.

### Variability between tool and spreadsheet based counting

To assess whether the tool contributes to lower variability in intra-observer measurements, in Fig. 2B we show histograms and BA plots comparing counts obtained via the tool or spreadsheet measurements using the same, ExP or NExP, observer, shown respectively left and right. Note that deviation is higher when compared to the intra-observer findings using the tool alone (previous paragraph). It appears that the tool has less effect (smaller deviation) to an ExP, whereas it seems to help reduce variability for NExP. This adheres to comments of NExP observers stating that when leaf numbers are high, and plant structure appears complex, it is hard to keep counting the leaves manually without visual reference resulting in frequent restarts of counting (even 3 times). We note that the tool retains visible the placed dots to precisely help visual memory. The same conclusions can be drawn from the statistical numbers shown in Table 1, however with slightly decreased agreement in the NExP observer.

All the results presented in the following refer to tool based annotations.

### Inter-observer variability

To assess inter-observer variability we selected one experienced observer as a reference and compared against other ExP and NExP observers (a total of 9), which allows us to be concise (e.g. by showing representative comparison pairs instead of all possible combinations). Although this approach does not take into account observation error of the reference observer, the chosen observer had the smallest intra-observer variation (see entry marked with a ‘[Reference observer]^a^’ in Table 1.)

Figure 3A and B visualize inter-observer agreement in the case of RPi and Canon, whereas Table 1 offers statistics. Overall we see that agreement is excellent independent of experience. At times experienced observers appear to disagree more particularly when resolution is higher. This is likely attributed to how experienced observers appreciate new leaf emergence and particularly if they are trained to see it or not.

### Influence of resolution on intra-observer variability

This variation among experienced observers becomes also evident when comparing the same observer and their annotations when resolution alters. The ExP observer (who is also the reference) tends to underestimate when resolution is lower. Whereas the NExP observer shows less under-estimation and higher agreement. It appears that NExP observers may miss young leaves independent of resolution (as they are not trained to see them) whereas the ExP observer misses them only on lower resolution.

### Influence of observer variation in longitudinal analysis

In Fig. 4 we show per-day average leaf count for each cultivar (i.e. averaging across replicates) when using annotations from different sets (and numbers) of observers for the RPi data. The top row refers to using a single ExP or NExP observer i.e. averaging within the population of each cultivar (panel A); whereas the middle row refers to a group of observers within their expertise, averaging first across observer annotations, and then across replicates (panel B). Panel C is similar to B but averages across all observers. The plots show average leaf count (within the population of each cultivar) and 1 standard deviation (shading) from the mean of the population. It is evident that given the effect size of the chosen cultivars, trends of average leaf count are expected even when using a single observer, albeit the ExP observer shows less variation. When combining observations across a group of observers trends still show even clearer and one may even argue that averaging across NExP tends to perform even better than a single NExP observer (compare panel B and A).

In Table 2 the results of the statistical ANOVA experiment are shown focusing only on the interaction term of interest (time*cultivar). We can see that in all cases the interaction is significant (*p* ≤ 0.05) confirming the visual findings of Fig. 4 and analyzed above. Note that although the smoothing effect is evident in the plots, when using more observers slightly increases the *p* value (decrease of the *F* score). This could be attributed to the fact that when using a single observer their behaviour (e.g. tendency to under-estimate) may be considered a fixed effect which is captured in the intercept, whereas using a population of observers (even of the same expertise) this may not be captured by the specification of the ANOVA model.

### Time results

Overall, we find that on average observers using the tool spent 48 min to annotate 130 plants for an average of 21 s per plant. Observers using the spreadsheet took on average 42 min. These findings were obtained by recording start and stop times of 5 observers in a controlled setting and provide aggregate timing information across an annotation task.

On the other hand, by keeping track of time when annotations were placed using the tool, more precise per leaf timing annotations were obtained (see “Methods”). Since this approach assumes that observers continuously label leaves, which may not hold if they take a break whilst labeling a plant, times greater than 200 s were considered outliers and were excluded from analysis.

Recording the time required to annotate a plant, we found that there is no statistical difference between experienced and non-experienced observers (*p* value 0.245). On average, within the 21 s required to annotate a plant, only 8.5s were used to actually complete the task. (In general, an annotator takes 1.10 ± 2.15 s per-leaf). We argue that annotators use the remaining time to assess how to annotate a plant and evaluate the quality of their own work. In fact, several annotators were double-checking their work after they finished to annotate all the leaves. We found this by analysing the timestamps recorded for each annotation. For some plants, the last annotation was placed after 40 min from the first one on the same image. Moreover, we also found no correlation between errors and time. Specifically, comparing the leaf count with the reference expert, the DiC is not affected over time.

### Simulating a citizen-powered study

Given the number of available observers on RPi (9 observers) and the *a priori* knowledge of their experience, it is of interest to explore: (i) the effects of using multiple observers for phenotyping by reducing their load (i.e. not having to annotate all images but a fraction of them) and consequently; (ii) the potential of using citizen-powered research platforms for phenotyping (where experience could be an unknown factor).

At first instance we wanted to simulate how many annotations we need to still maintain the phenotyping findings of the previous section: i.e. that there is an effect between time and genotype in the ANOVA setup. For this purpose we set-up a Monte Carlo simulation study that at each trial randomly draws a sampling matrix with *K* observations per time point. For example, for two observations per time point, this matrix has *K* = 2 ones per row (a row is an observation) for a total of 260 ones (the rest being zeros). The placement of ones select from which annotator an observation is obtained for this time point. For more than 1 annotation per time point (i.e. plant image), annotations across observers are averaged.

We varied *K* = 1, 2, 3 drawing from all available annotators (*n* = 9) or only from experienced (*n* = 5) or non-experienced observers (*n* = 4) to inspect the influence of mixing experience in annotations in the overall result. At each trial we run the ANOVA experiment and record the *p* value of the interaction term (time*cultivar). We draw 500 trials for each variation of setup (*K* and the observer groups) and finally obtain summary statistics of the distribution of the *p* values among the 500 trials, namely minimum, maximum, mean, standard deviation, and kurtosis (a notion of symmetry and normality).

Table 3 reports the findings of this study. Overall we see that at no point, independently of the number of annotations used or the experience of observers, the *p* value is not statistically significant (the max *p* value is always below the significance threshold). This is telling since even 1 annotation is enough for the effect size observed in these cultivars. With 1 annotation per time point, with 9 observers this would have an effect of reducing annotation effort per-observer to 11.1% of the dataset (i.e. 14–15 plants per each observer). As expected the more observers the better; but sampling only from experienced observers did not necessarily outperform sampling only from non-experienced ones. Given the leptokurtic characteristic of these distributions (high kurtosis), the distributions are highly peaked around the mean with values concentrating around these. Overall, while the max indicates the worst expected result, results around the mean are to be expected as more typical.

### Results from the citizen-powered study

The study was launched on May 1st 2017, and by June 1st, approximately 5000 user annotations were available on a dataset of 1248 images, including the 130 RPi images used in this paper, with each image having at least 3 user annotations. Data were extracted from the Zooniverse database and a similar statistical analysis as to the one outlined above was carried out.

Of the 5000 annotations 4 Zooniverse users were responsible for annotating close to 10% of the data, as we can see in Fig. 5A. Most users contribute few annotations (long tail to the right), and not surprisingly most of the users are logged in (shown as black stem line without a marker in Fig. 5A), which implies that they are frequent contributors to the platform.

Of particular interest is to explore if the self-reported confidence (answering the question on whether they believe they have annotated all leaves) relates to the spread of leaf counts among users for each plant. Figure 5B shows a two dimensional histogram of the per-plant standard deviation of the reported leaf count among the users with none referring to 0 standard deviation (i.e. annotations agree fully) and the average confidence (averaging the confidence question) for each plant of the 130 used in this study. An average of 3 shows high confidence (y-axis) versus an average of 1 low confidence (y-axis). Color encodes probability of occurrence. Users tend to agree with each other and their self reporting of confidence appears to be consistent with their spread in counting leaves, since the upper left quadrant sums to approximately 70% of occurrences.

We then estimated a consensus citizen by averaging counts across the annotated counts for each plant. We compared this consensus against the reference observer (from our controlled study) and a random single selection of counts, which can be seen as selecting one count per plant out of the 3 citizen provided counts (shorthanded as sing. random in Table 1). The results of this analysis are shown in Fig. 5C and D respectively. We see what there is some variability among the reference observer and consensus citizen (Fig. 5C), with the latter underestimating counts (see also related entries of DiC in Table 1). On the other hand variability appears to be smaller within citizens (c.f. Fig. 5D and entries in Table 1).

Admittedly of most interest is to see if plain citizens can be used for actual phenotyping. We use the counts of the consensus citizen and plot as previously average (and one standard deviation) per cultivar counts as a function of time in Fig. 4D. We can see that this plot closely resembles the others and particularly the one of using only non-experienced observers in our controlled study. Equally the corresponding ANOVA experiment (last row in Table 2) shows exactly the same findings since using the consensus citizen counts yields a *p* value still statistically significant, albeit larger compared to the one of the controlled experiment. However, a key difference between the two exists: in our controlled study all observers rated all images, so perhaps fixed effects of each observer may be captured in the intercept. Instead in the citizen experiment all counts come from a large pool of observers. In fact, when we compare the *p* value of the consensus citizen (*p* = 0.0014) it is within the min-max bounds we find in our simulated study reported in Table 3.

Post-hoc, i.e. knowing that citizens under-estimate, under-estimation reaches 0 if we use the maximum across annotated counts (instead of average), and several other metrics improve including the *p* value of the ANOVA. In Tables 1 and 2 this is shown as consensus (max).

### Variability between algorithmic leaf count and experts

In addition to manual counting, we also tested a well-known leaf counting algorithm [15, 21] to assess whether algorithm error is within (or outside) human variation.

For this experiment, we used the plant images in [21], with annotations performed by experts not involved in other aspects of this study. Overall, this dataset contains 1248 individual images of plants, taken from five different cultivars (*col-0*, *pgm*, *ein2.1*, *ctr*, and *adh1*). Specifically, images of *ctr*, *adh1*, and *ein2.1* cultivars were used as training set (728 images in total), whereas the images of *pgm* and *col-0* cultivars, which were also used in this study, were employed as testing set (130 images in total). From the training images, we learned a plant descriptor that derives image features and the projected leaf area to learn a non-linear model to predict the leaf count. It is noteworthy that the training set contains cultivars not included in the testing set, which makes this learning protocol the most stringent condition as the algorithm has never seen the mutants. After the model was trained, we calculated the evaluation metrics in [21] in the training (728 images) and testing sets (130 images). In addition, since the expert observer that labeled the images used to train the algorithm was not part of this study, we also computed the disagreement between this expert and the reference observer used throughout this study.

As shown in Table 4, the algorithm learns well (agreement between algorithm and annotator on the 728 training images the algorithm was trained on). When predicting counts on the 130 test images, the algorithm performs slightly worse when compared with the same annotator involved in labeling the training set (middle column). However, we can see that the algorithm is within inter-observer variability which compares two expert annotators (last column in Table 4). While on average the algorithm predicts the correct leaf count on some images (mean close to zero) it appears that it is over- or under-estimating counts on some, which explains the high standard deviation and high MSE. We note that here the algorithm carries two sources of variation (error): one of the annotator and one of the learning process itself. The latter can be minimized, but the former unfortunately is harder to do so unless a mixture of annotators is used.

## Discussion and conclusion

In the following, we discuss the findings of our study, where we investigated observer variability for an annotation task being deliberately chosen to be simple to understand and perform for human annotators. Clearly, not all of these findings generalize to all (possible) human annotation tasks. Findings on ‘negative effects’, i.e. factors increasing annotator variability, like fatigue, lack of suitable annotation tools etc. can be expected to be also present for harder annotation tasks being more challenging for humans. They are expected to generalize well. However, ‘positive effects’, e.g. observed discriminative power of human annotations for the investigated task, cannot as easily be generalized to other, especially more difficult tasks.

In this study, we showed that intra-observer variability remains low with experienced observers, but non-experienced ones tend to vary more in their second repeat reading using a visualization tool. Our annotation tool helps to retain mental memory and to reduce fatigue overall lessening the potential for errors when plants become larger and have more leaves. At the same time we showed that higher image resolution helps, but not always with the same effect: higher resolution aids the experienced user to find more of the smaller leaves, but non-experienced ones missed them more often independently of resolution. Inter-observer variability is not significantly greater than intra-observer variability. Overall observers tend to be within plus/minus one leaf almost 80% of the time.

This agreement seems appealing but it might be random in nature and we explored if it affects the use of observers in actually identifying group differences in longitudinal counts. Repeat statistical tests showed that, when we use one or more experienced or non-experienced observers, we still come to the same statistical conclusion using an ANOVA test on the same longitudinal cultivar comparison: we find, as expected, differences in trends between *col-0* and *pgm* as reported previously on the same data [21]. Whether we use only experienced or non-experienced observers has minimal effects on the statistical inference of the test.

Encouraging are the investigations using simulated and real data from citizen-powered experiments. In real experiments we cannot ensure the composition (in expertise) of the participating users and neither we can assume that the same user will annotate all the data. However, our analysis on simulated data (where we can control the composition) showed that having even 1 annotation per plant can be sufficient to arrive to the same statistical conclusion (differences in cultivar trends) but of course having more is better, reducing variation. These findings held also in the real citizen-powered experiment based on the Zooniverse platform. Leaf counting based on algorithms while showing promise and progress does not yet meet human performance necessitating further investigation in the area; thankfully, collation studies [14] and challenges (e.g. the counting challenge of the CVPPP workshop series https://www.plant-phenotyping.org/CVPPP2017-challenge) on open data [11] will help advance the state-of-the-art.

This paper points to several potential areas for further research. Variability will be present in annotations and we can either obtain a better consensus, learn to ignore this variability, or alter the annotation task to minimize variability. In this study consensus was obtained through averaging across annotations and treating time points independently, but alternative mechanisms can be used to establish more consistent longitudinal counts. For example, one can adopt several other consensus approaches that are data-agnostic [48] or if we assume that leaves always emerge or remain the same in succession of images but cannot disappear, consensus can be derived using a dynamic filtering approach. Alternatively, machine learning algorithms can be used to learn directly from such repeated and imprecise (in machine learning speak: noisy) annotations potentially also obtaining consensus estimates which should also help eliminate observer bias. However, in machine learning much effort has been devoted to noisy annotations in classification tasks [37, 38] but in regression is a yet unexplored area. A more radical approach, is to alter the design of the annotation task completely: for example, users can be shown pairs of images and can be asked to identify only ‘new’ leaves (if any at all). Irrespective of the design of the annotation task, minimizing the amount of data requiring annotation by selectively displaying (to the observers/annotators) only images that do need annotation is always desirable. This has strong links to active (machine) learning [49] which displays images that are the most informative from a machine learning perspective. Integrating this may be possible within a controlled lab annotation platform (as for example with the CellProfiler [49] software^3^) but doing so in Zooniverse is not straightforward as images used in the work-flow cannot be altered on the fly and a customized platform would be required.

Considering all these findings we can conclusively argue that while there is some variability among observers it is minimal when evaluating quantitative traits like counting objects, even of very different sizes. For the group (cultivar) effect sizes observed here this variability had no effect in statistical inference. At the same time common citizens, empowered by easy to use platforms, can greatly assist the effort of annotating images; at least, when the overall task is broken down in elementary sub-tasks generally doable even by non-experts without detailed explanations. Then common citizens can be used to provide annotations and drive phenotypic analysis. Such annotations help to develop and evaluate automated algorithms and allow to train machine learning-based solutions. Using such platforms a higher annotation throughput can be met than perhaps available locally in a lab, reducing significantly annotation effort.^4^ It is time to consider how we can motivate the participation of citizens and design annotation tasks that can provide data of sufficient quality for other phenotyping tasks. This will have not only an effect on phenotyping but also on introducing this societally important problem to the broad public.

## Footnotes

1 This more closely emulates how experts rate data with visual scales in the field since there is an inherent assumption that previous ratings and images of the scene are not used as reference.

2 More information at http://phenotiki.com.

3 This is planned to be made available in Phenotiki in mid 2018 for the counting module.

4 We emphasize that Zooniverse is not an annotation platform per se and any workflow presented should have a strong ethical and reward mechanism to be accepted as a Zooniverse project. For tasks with a demanding rate and purely annotation objective gamification and crowdsourcing should be selected.
